# Optimizing the Sensitivity of Biological Particle Detectors through Atmospheric Particle Analysis According to Climatic Characteristics in South Korea

**DOI:** 10.3390/s22093374

**Published:** 2022-04-28

**Authors:** Hyunsoo Seo, Kibong Choi, Young-Su Jeong

**Affiliations:** Chem-Bio Technology Center, Agency for Defense Development, Daejeon 34186, Korea; skhsjwjw@add.re.kr (H.S.); kibongchoi@add.re.kr (K.C.)

**Keywords:** biological aerosol detection, real-time analysis, laser-induced fluorescence, particle counting, single-particle analysis

## Abstract

Biological agents used in biological warfare or bioterrorism are also present in bioaerosols. Prompt identification of a biological weapon and its characteristics is necessary. Herein, we optimized an environmentally adaptive detection algorithm that can better reflect changes in the complex South Korean environment than the current models. The algorithm distinguished between normal and biological particles using a laser-induced fluorescence-based biological particle detector capable of real-time measurements and size classification. We ensured that the algorithm operated with minimal false alarms in any environment by training based on experimental data acquired from an area where rainfall, snow, fog and mist, Asian dust, and water waves on the beach occur. To prevent time and money wastage due to false alarms, the detection performance for each level of sensitivity was examined to enable the selection of multiple sensitivities according to the background, and the appropriate level of sensitivity for the climate was determined. The basic sensitivity was set more conservatively than before, with a 3% alarm rate at 20 agent-containing particles per liter of air (ACPLA) and a 100% alarm rate at 63 ACPLA. The reliability was increased by optimizing five variables. False alarms did not occur in situations where no alarm was unnecessary.

## 1. Introduction

Recently, interest in bioaerosols is growing worldwide as people directly experience the dangers and damage of unknown new infectious diseases such as coronavirus disease 2019, which developed into a pandemic. Bioaerosols refer to biological factors such as microorganisms, (i.e., viruses, bacteria, and fungi), spores, pollen, and droplets that exist in a dispersed state in indoor and outdoor air environments as fine particles [1]. Biological agents used in biological warfare or bioterrorism are also present in bioaerosols. Since most of them are colorless and odorless and are dispersed in the air (causing extensive damage to numerous people in a non-targeted manner), it is necessary to quickly determine the presence of a biological weapon and its characteristics [2,3]. In particular, to prepare for biological warfare in South Korea, it is necessary to acquire data reflecting complicated and complex environmental changes due to its geographic location and varied topography. The peninsular environment in South Korea is largely characterized by recurrent Asian dust in spring, the rainy season in summer, and snowfall in winter. Although regional particulate matter (PM_10_; particles with diameters less than 10 µm) and fine particulate matter (PM_2.5_; particles with diameters less than 2.5 µm) trends have been studied in the Korean Peninsula [4], and their chemical properties have been analyzed [5], relatively little research has been conducted to characterize bioaerosols in the South Korean environment, with limited quantitative estimations of aerosols due to complex interactions [6]. Therefore, the aim of this study was to quantitatively analyze the distribution of bioaerosol concentrations based on climatic variations in the Korean Peninsula.

Devices like an optical particle counter (which is based on the principle that the size of a scattered light signal is proportional to the particle sizes) and an aerodynamic particle sizer (APS; used to measure aerodynamic properties that vary depending on the characteristics of individual particles with the time-of-flight method) are used to detect significant increases in the concentrations of aerosol in real-time [7]. Because these particle analyzers generally measure scattered light, they cannot measure a fluorescence signal that is far smaller than the signal of scattered light, and only particle size information can be obtained. Therefore, while an ultraviolet APS (UV-APS; a fluorescence analyzer) can be used for bioaerosol analysis, it is impossible to control the measurement sensitivity, limiting bioaerosol-monitoring surveillance in a complex environment [8]. The use of a fluorometer may also be considered, but a disadvantage exists in that it is impossible to distinguish the size of particles produced in the 2–10 µm range, which causes significant damage with a biological weapon in real-time [9].

Therefore, a biological particle detection technology enabling real-time measurement and size classification, and an environmentally adaptive detection algorithm have been developed [10,11,12]. In this study, we intended to improve the laser-induced fluorescence (LIF)-based biological particle detection technology that was developed in a previous study to ensure its application without false detection in various environments. Real-time changes in the complex South Korean environment were measured by simultaneously measuring scattered light and fluorescence was used to analyze related data and provide representative particle information. Finally, the basic parameters of the detection algorithm were optimized for the South Korean environment by analyzing light scattering and fluorescent particles in various climates. Here, we obtained more accurate results by comparing our current data with the findings of previous studies, which did not consider the particle characteristics under each climate. Therefore, the characteristics of ordinary particles, including those of biological particles (which can be confused with the characteristics of meaningful biological weapons), were classified, and the detection algorithm was adjusted to issue an alarm in the environment only where necessary. Based on experimental data gathered in various regions and climates, the environmentally adaptive-detection algorithm was trained and its detection performance was optimized according to the sensitivity level determined in the corresponding climate. This study is significant in that it measures bioaerosol, which has become a problem recently, in real-time. Our findings make it possible to increase the reliability of the biological particle detector and adjust the sensitivity fluidly according to the South Korean environment.

## 2. Materials and Methods

### 2.1. Optics Design

The LIF-based biological particle detector used in this study has an air-intake rate of 1 L/min and measured scattered light and fluorescence generated by the interaction between the ultraviolet (UV) light beam and the air particles. A UV-light-emitting diode (LEUVA6600HF00, LG Innotek, Seoul, Korea) with a central wavelength of 278 nm was used as the UV light source. The configuration of the optical module was described previously [9]. The signal collected from the optical cell and output from the optical chamber was separated into scattered light and fluorescence with a beam splitter (Beam Splitter, FF310-Di01, Semrock, Rochester, NY, USA). The weak scattered light and fluorescence signals were amplified through a photomultiplier tube (PMT) detector (H10721P-110, Hamamatsu Photonics K.K., Shizuoka, Japan) to analyze the particle sizes and the characteristics of fluorescence induced by the biological particles. The scattered light and fluorescence signals collected through the PMT sensor were converted into digital signals for use in detection algorithms. The scattered light and fluorescence results, counted as digital signals, were transmitted to a microcontroller unit (MCU; STM32F420, STMicroelectronics, Geneva, Switzerland). Since the counting quantity reflected the pulse width in the MCU, the particle signals were classified as small, medium, large, and under particles. Effective particles were entered as parameters for the detection algorithm. Because the particles in biological weapons are approximately 1–10 µm in diameter, the blocking threshold for the particle size was set within the corresponding range. That threshold was determined experimentally in a previous study, and adjustments were made to set the diameters of small, medium, and large particles at 0.84–2 µm, 2–5 µm, and 5–11 µm, respectively [12]. 

### 2.2. Algorithm

The signal processing performed in this study enabled particle detection only when the sizes of the scattered light and fluorescence signals were the same. The sizes and numbers of small, medium, and large scattered light and fluorescence signals were acquired every 10 s. As the most basic variables, the sums of the scattered light and fluorescence signals (measured regardless of size) were obtained, and the ratio of fluorescent particles to the total particles was also determined to identify the average distributions of the fluorescence index (defined as the sum of the fluorescence signal/sum of the scattered light signal) in various environments. The fluorescence index was calculated by accumulating data for 3 min, and even if an abnormality was found, sufficient time was allowed until the abnormal data value was reflected in the average value to issue an accurate alarm. The threshold of the fluorescence index was adjusted according to the sums of the scattered light and fluorescence signals. High scattered light and fluorescence sums were considered to reflect a polluted environment, and the sensitivity was adjusted to low. Low scattered light and fluorescence sums were considered to reflect a clean environment, and the sensitivity was adjusted to high. The scattered light: fluorescence-distribution weight values were set to prepare different alarm criteria according to the corresponding observed ratios and particle distributions.

**Figure 1:**
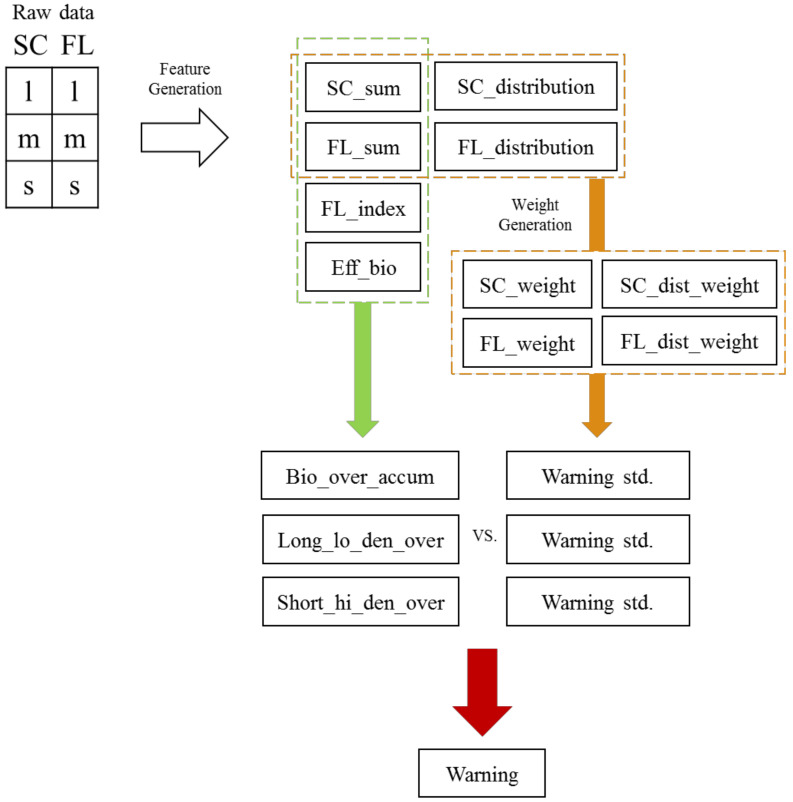
Parameters and design of the detection algorithm.

The artificial sizes of biological weapons are medium and large, and the smaller the small/large is, the more dangerous it is to the environment. In this case, the sensitivity can be increased for seamless detection. The inclusion of 36 variables, which affect one another, could increase the reliability of the algorithm. In this study, only 17 variables that required optimization through learning about the environment were considered (Figure 1). As a result, a final alarm was issued if the sum of the alert variables was greater than or equal to the alert reference variable. The reference variable for the final alarm had a maximum value of 6, as it was based on the 1-min average of data acquired every 10 s. The alarm variables included a relative concentration alarm, a long-term low-concentration alarm, and a short-term high-concentration alarm. Each alarm reference variable was calculated as a function of the fluorescence index, scattered light sum, and effective biological particles (defined as the fluorescence sum − (the scattered light sum × the fluorescence index)). 

The relative concentration alarm served as an index indicating the air-pollution level measured in the last min. The final alarm reference variable was determined by adding the scattered light and fluorescence weights to this value. For this variable, a confidence interval was set as the standard deviation of the effective biological particle, assuming a normal distribution in the change of biological particles in the background to prevent the alarm reference variable from being affected by the polluted environment value when abnormal symptoms occurred. An experimentally reasonable confidence interval was established, which was initially set at 80%, corresponding to a value of 1.28. The confidence interval was designed so that the value could be modified through later experiments. Because it was difficult to detect slowly increasing particle numbers using only the relative concentration alarm, a long-term low concentration alarm was also designed. This alarm was issued when the value of the effective biological particle was stably maintained above the reference variable. Even if the long-term low concentration was exceeded, if a certain fluorescence index was not reached, an alarm was not generated, thereby, reducing the occurrence of false alarms. Furthermore, because it was difficult to detect biological particles exceeding the short time limit, a short-term high-concentration alarm was designed to be issued when the concentration of the effective biological particle was maintained above the reference variable for a short period of time. The final alarm was set to sound when the reference variable for at least one of these three types of alarms was met.

## 3. Results and Discussion

**Figure 2:**
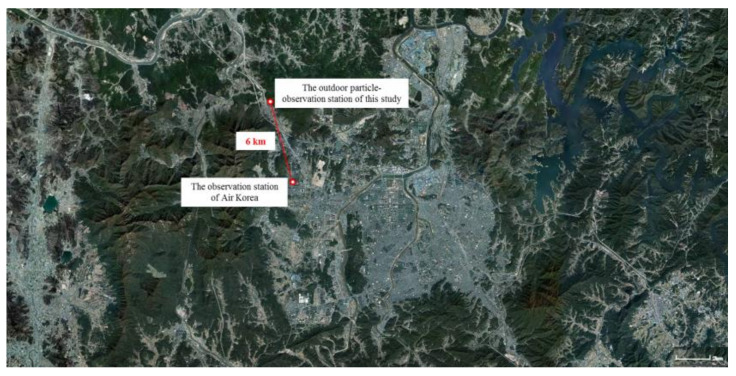
The location of the observation station of Air Korea and the outdoor particle observation station of the biological particle detector used in this study.

First, a comparison with Air Korea data was conducted to confirm whether the particle measurements in this study were reliable. Air Korea is a website that discloses real-time data from the National Ambient Air Quality Monitoring Information System (Korea Environment Corporation) and provides environmental information such as PM_10_ and PM_2.5_ data for different regions [13]. Air Korea provides information for airborne dust particles less than 10 µm in diameter during each hour. These data were compared with the scattered light sums (counts/L) determined in this study, which comprised the sums of small, medium, and large scattered light signals detected by our biological particle detector. The observation station of Air Korea was located in Noeun-dong, Yuseong-gu, Daejeon, and the outdoor particle observation station for this study (Sunam-dong, Yuseong-gu, Daejeon) was located about 6 km away (Figure 2). The observational data were collected inside the wind tunnel for six consecutive days from 16 February to 22 February 2021. Although the biological particle detector obtained real-time measurements every 10 s, the Air Korea data represented values collected over 1 h. Therefore, the graphs were plotted at 1 h intervals for comparison and analysis purposes.

**Figure 3:**
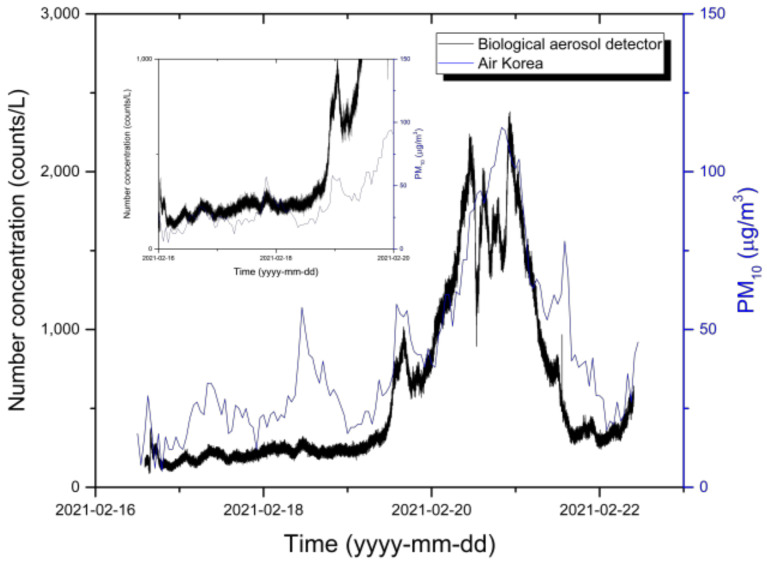
Comparison of Air Korea data with the particle measurement results of our biological particle detector to verify its reliability. The X-axis represents the measurement period, and the Y-axis represents the measured particle concentrations, respectively. The blue line represents the particle observation results from Air Korea, and the black line represents the particle concentrations (counts/L) of the biological particle detector.

Figure 3 compares the hourly PM_10_ data (µg/m^3^) from Air Korea and the scattered light sums (counts/L) of the biological particle detector. The PM_10_ concentrations from Air Korea gradually increased over time for 5 days, reaching a maximum of 114 µg/m^3^ at 21:00 on 20 February 2021, and then gradually decreased again. During this period, the average concentration was 41 µg/m^3^, with a minimum concentration of 5 µg/m^3^. As with Air Korea, the scattered light sum of the biological particle detector gradually increased for 5 days, showing a maximum value of 2381 counts/L at 22:00 on 20 February 2021, and then gradually decreasing again. The average concentration was 226 counts/L, with a minimum of 87 counts/L. These results exhibited similar overall tendencies. To confirm the observed trend for low concentrations, the Y-axis for the period from 16 February to 20 February 2021 was set to 1000 counts/L (or 150 µg/m^3^) for comparison purposes. The trend of PM_10_ increasing to 57 µg/m^3^ at 11:00 on 18 February 2021 and decreasing to 33 µg/m^3^ at 17:00 was consistent with the trend of the scattered light sum on the biological particle detector decreasing from 324 to 186 counts/L from 12:00 to 17:00 on 18 February 2021. Such results indicated that the trends in the overall particle observation results of the biological particle detector and Air Korea data were consistent during the observation period. This finding confirmed that the biological particle detector used in this study accurately observed the external environment. Therefore, we analyzed light scattering and fluorescent particles in the South Korean environment.

### 3.1. Particle Analysis in Different Climates

Data in various climates were collected to analyze scattered and fluorescent particles in the South Korean environment. The climates included rainfall, snow, fog, mist, water waves, and Asian dust. Because meteorological conditions such as rainfall, snow, fog, and mist may show different behavior even in similar climates, two cases were considered for each parameter. In each case, the measurement period and climate are summarized in Table 1, where the main climate time in the measurement period is separated as the “time in the climate.” This time is indicated by a blue solid line (in a different color from the background) in the graph. In the graph, the meaning of scattered light and fluorescence sums refers to the input data for effective particles and effective-biological particles, respectively. The sums reflect the small/middle/large-particle light scatter and fluorescence inputs every 10 s. Because the intake of the biological particle detector is 1 liter per minute, the value multiplied by six represents 10 s of data. The characteristics of general and biological particles in the South Korean environment were analyzed by determining the characteristics of particles in each climate using a biological particle detector, and the alarm rate was improved to optimize the algorithm.

#### 3.1.1. Rainfall

During the rainfall period in Case 1, the maximum scattered light sum was 6354 counts/L, the minimum was 210 counts/L, the mean was 1548 counts/L, and the standard deviation was 1576 counts/L. The maximum fluorescence sum was 390 counts/L, the minimum was 0 counts/L, the mean was 8.5 counts/L, and the standard deviation was 22.67 counts/L. The scattering: fluorescence ratio (%) was 0.96, and the relative concentration was exceeded 1192 times, with the final alarm issued 151 times. During the rainfall period in Case 2, the maximum scattered light sum was 3312 counts/L, the minimum was 204 counts/L, the mean was 1597 counts/L, and the standard deviation was 820 counts/L. The maximum fluorescence sum was 120 counts/L, the minimum was 0 counts/L, the mean was 4.8 counts/L, and the standard deviation was 15.30 counts/L. The scattering: fluorescence ratio (%) was 0.69, and the relative concentration was exceeded 151 times, with the final alarm issued once.

**Figure 4:**
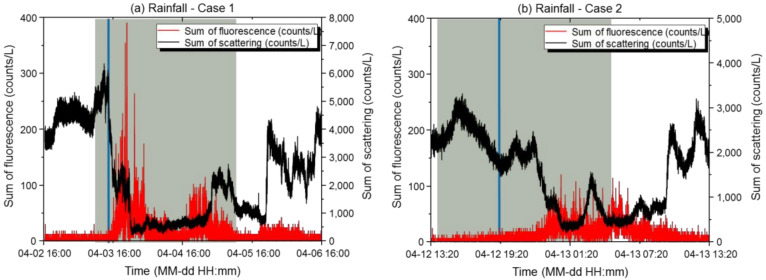
Results of scattered light and fluorescence measurements with the biological particle detector during rainfall. (**a**,**b**) Time-dependent concentrations of light scattering and fluorescent particles in Case 1 (**a**) and Case 2 (**b**). The X-axis represents the measurement period, and the Y-axis represents the measured particle concentrations. The black line represents the scattered light sum (counts/L), the red line represents the fluorescence sum (counts/L), the blue line represents the time of maximum rainfall, and the green line represents the time period when the meteorological condition occurred during the measurement period.

Figure 4 shows the effective numbers of light scattering and fluorescent particles versus time at the basic sensitivity. In all cases, the scattered light sum increased rapidly as the rain started to fall and tended to gradually decrease thereafter. After the rainfall stopped, the scattered light sum increased due to the particles being scattered again. At the maximum rainfall, the particle number concentration increased under the influence of water droplets, and then the scattered light sum decreased as the rainfall continued. The maximum fluorescence sum occurred 5 to 6 h after the maximum rainfall and then decreased slightly immediately after the rain stopped. The humidity remained very high at 75% during the period (13 April 2021) even after the rain stopped. The fluorescence sum was maintained even after rain as new fluorescent particles were emitted from the active biota (fungus, moss, rocks, and vegetation) on wet surfaces following rain. High temperatures during rainfall may affect the activity levels of biological particles, thereby, increasing the emission of fluorescent particles [14,15]. In Case 1 (3 April 2021), the fluorescence sum increased by up to 80-fold in the afternoon as rain fell in the morning and the temperature remained high. In Case 2 (12 April 2021), the fluorescence sum increased only by up to 1.3-fold as rain fell at dawn and the temperature remained low, suggesting that temperature affected the distribution of fluorescent particles. Furthermore, by comparing the maximum rainfall for each case, we found that the scattered light sum was 1.9-fold higher and that the fluorescent light sum was 1.1-fold higher than the corresponding values found with weak rain. This is because biological particles bounce from surfaces and are newly emitted more frequently in heavy rain. These environmental characteristics can affect the alarm rate and false detection rate, resulting in wasted military efforts. The tendencies of the ratios of small, medium, and large light scattering and fluorescent particles during rainfall are the same. The proportion of small light scattering particles increased due to raindrops when the rainfall was at its maximum, and the proportions of medium and large light scattering particles decreased. As the rainfall stopped, the proportion of small light scattering particles increased due to smaller raindrops and re-scattering due to the drying of wet dust. The ratio of small fluorescent particles tended to increase when rain kept falling, which was thought to reflect the phenomenon of biological particles bouncing on the soil and vegetation surface. The proportions of medium and large fluorescent particles decreased as the rain kept falling, showing the same trend observed with the medium and large light scattering particles, for the same reason. When rain fell in the afternoon, the proportion of medium fluorescent particles increased, in particular, as mold was activated when the humidity was maintained (12 April 2021; weak rain).

As described above, the alarms were issued mainly when the scattered light sum was very low (≤100 counts/L). The scattered light sum correlated with the rainfall situation; thus, the alarms were issued 8834 times and 3141 times, respectively, before optimization. Because the mean effective number of biological particles and confidence interval is small in a clean environment, false alarms increase even with a slight increase in biological particles. Thus, a reference variable for determining exceedance was set. During rainfall, alarms were issued because many biological particles with a small scattered light sum were detected. Therefore, during rainfall, the sensitivity should be adjusted to low by maintaining the reference variable at the maximum value of “5”. In addition, a long-term low-concentration alarm may be generated during rainfall. In a normal climate, no alarm is issued unless a certain fluorescence index is reached, even when the criteria for the long-term low concentration standard are met. With an increase in biological particles due to rain, the fluorescence index tends to increase, so it seems appropriate to set the sensitivity to low during rainfall. The alarm rates before adjusting the sensitivity to reflect the South Korean environment were 8834 and 3141 for Cases 1 and 2, respectively. However, after optimizing the basic sensitivity, the alarm rates decreased to 151 and 1, respectively. Therefore, the false alarm rates were reduced by up to 99.98%. In particular, when operating at low sensitivity, 0 alarms were issued in both Cases 1 and 2.

#### 3.1.2. Snow

**Figure 5:**
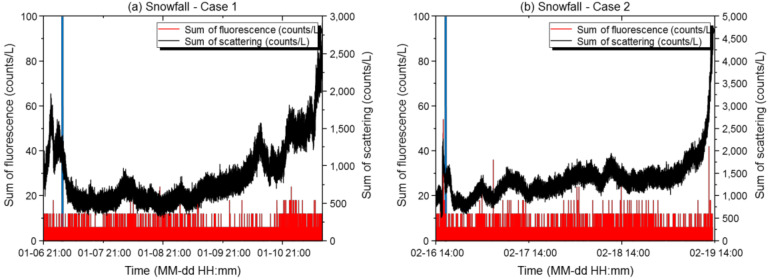
Results of scattered light and fluorescence measurements of the biological particle detector during snow. (**a**,**b**) Time-dependent concentrations of scattered light and fluorescent particles in Case 1 (**a**) and Case 2 (**b**). The X-axis represents the measurement period, and the Y-axis represents the measured particle concentrations. The black line represents the scattered light sum (counts/L), the red line represents the fluorescence sum (counts/L), and the blue line represents the time of maximum snowfall.

During the snow period in Case 1, the maximum scattered light sum was 2910 counts/L, the minimum was 312 counts/L, the mean was 857 counts/L, and the standard deviation was 400 counts/L. The maximum fluorescence sum was 24 counts/L, the minimum was 0 counts/L, the mean was 0.8 counts/L, and the standard deviation was 2.33 counts/L. The scattering: fluorescence ratio (%) was 0.19, and the relative concentration was exceeded 0 times, with the final alarm issued 0 times. During the snow period in Case 2, the maximum scattered light sum was 4770 counts/L, the minimum was 522 counts/L, the mean was 1360 counts/L, and the standard deviation was 424 counts/L. The maximum fluorescence sum was 180 counts/L, the minimum was 0 counts/L, the mean was 1.1 counts/L, and the standard deviation was 3.04 counts/L. The scattering: fluorescence ratio (%) was 0.26, and the relative concentration was exceeded 7 times, with the final alarm issued 0 times. Figure 5 shows the effective number of scattered light and fluorescent particles versus time at the basic sensitivity setting.

The snow condition was characterized by low pressure and high winds, where the average particle count was lower than that during rainfall, where the number of particles detected tended to be low. The average humidity during both measurement periods (Cases 1 and 2) was as low as 63%, which gradually decreased after the maximum snowfall to a minimum of 40%. Therefore, unlike during rainfall, the role of particle nucleation decreased due to low humidity. In both cases, the scattered light sum increased during the initial snowfall and then decreased sharply. It then gradually increased toward the end of the snowfall, followed by a sharp increase again during late snowfall. The initial particles were removed by inertial collision with the snowflakes, and the gravitational settling mechanism also acted upon them during the maximum snowfall, which rapidly decreased the number of particles detected. During late snowfall, particles in the atmosphere were settled by gravitational settling, after which the particles increased rapidly as the snow subsided and the particles became resuspended as the temperature rose. After snowfall (period after the blue solid line in Figure 5), the temperature and humidity gradually increased, while the accumulated snow gradually melted and the particles re-scattered with dust, thereby, increasing the scattered light sum [16,17]. Fluorescence remained stable before and after the snowfall, and no fungal activation was observed due to the relatively low temperature (compared to those in other seasons). During snowfall, the fluorescence index was very low in Cases 1 and 2 (0.0009 and 0.0010, respectively), and 0 alarms were issued both before and after optimization. Compared to the rainfall environment, both the scattered light and fluorescence sums were low during snowfall, as the environment was not conducive to a rebouncing phenomenon. Therefore, the biological particle detector could be operated at the basic sensitivity. The alarm rates before adjusting the sensitivity to reflect the South Korean environment for Cases 1 and 2 were 296 and 0, respectively. Operating the biological particle detector at the optimized basic sensitivity reduced both false alarm rates to 0 (up to a 100% decrease).

#### 3.1.3. Fog and Mist

**Figure 6:**
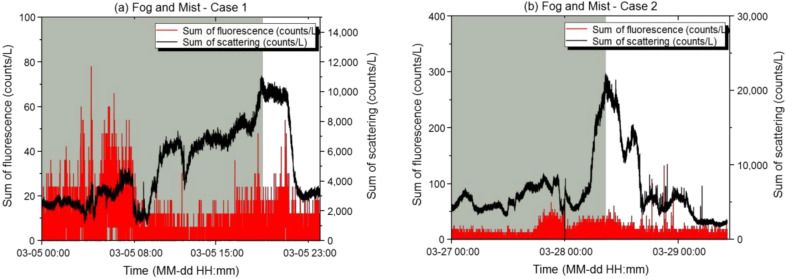
Results of light scattering and fluorescence measurements of the biological particle detector during fog and mist. (**a**,**b**) Time-dependent concentrations of light scattering and fluorescent particles in Case 1 (**a**) and Case 2 (**b**). The X-axis represents the measurement period, and the Y-axis represents the measured particle concentrations. The black line represents the scattered light sum (counts/L), the red line represents the fluorescence sum (counts/L), and the green line represents the time period when the meteorological condition occurred during the measurement period.

Fog is a state in which water vapor near the surface of the earth is condensed and floats in a cloudy state. Fog refers to conditions where the visibility is <1 km, and mist refers to conditions where the visibility is between 1 and 10 km. The fog and mist data from the automated surface observing systems were used. During the fog and mist periods in Case 1, the maximum scattered light sum was 11,064 counts/L, the minimum was 1086 counts/L, the mean was 4762 counts/L, and the standard deviation was 2580 counts/L. The maximum fluorescence sum was 78 counts/L, the minimum was 0 counts/L, the mean was 3.6 counts/L, and the standard deviation was 8.77 counts/L. The scattering: fluorescence ratio (%) was 0.15, and the relative concentration was exceeded 77 times, with the final alarm being issued 0 times. During the fog and mist periods in Case 2, the maximum scattered light sum was 18,886 counts/L, the minimum was 3114 counts/L, the mean was 6605 counts/L, and the standard deviation was 1374 counts/L. The maximum fluorescence sum was 72 counts/L, the minimum was 0 counts/L, the mean was 3.8 counts/L, and the standard deviation was 8.31 counts/L. The scattering: fluorescence ratio (%) was 0.11, and the relative concentration was exceeded 101 times, with the final alarm being issued 0 times. Figure 6 shows the effective number of light scattering and fluorescent particles versus time at the basic sensitivity setting.

Unlike the particle behaviors observed during rainfall and snow, the increases and decreases in light scattering and fluorescent particles showed the same trends during fog and mist. The scattered light sum due to moisture increased and the number of fluorescent particles increased, as the microscopic biological particles acted as nuclei. The fluorescence sums were similar in each case (3 counts/L). However, during afternoon fog (Case 2), the scattered light sum was twice as high as that during morning fog, with a lower fluorescence index. Using our algorithm, the fluorescence index was determined based on different scattered light sum criteria for the morning and afternoon. However, no false alarms occurred. In addition, 2–3-fold more fluorescent particles were detected during fog than in the absence of fog. In particular, a difference of up to 4-fold or more was found in the number of small fluorescent particles. Therefore, we examined whether the algorithm weights worked properly even in the event of a sudden environmental change. Because fog may contain various particle concentrations (with scattered light sums ranging from 6000 counts/L to 25,000 counts/L), the reference variable for the fluorescence index must be set accurately, according to the scattered light sum. As an alarm was issued with the original settings, the scattered light weight was adjusted, and the reference variable for the fluorescence index was adjusted higher than that used for the previous variable, according to the range of the scattered light sum (6000 counts/L). Accordingly, the size that received only a weight of −1 was weighted from −1 to 0.5, and the range of the lowest scattered light sum among the reference variables of the fluorescence index was adjusted to reduce false alarms. In contrast to rainfall, fog and mist showed parallel increases or decreases in the scattered light and fluorescence sums, and no alarm was issued. Therefore, the biological particle detector should be operated by maintaining the basic sensitivity setting during fog or mist.

#### 3.1.4. Asian Dust (Yellow Dust)

**Figure 7:**
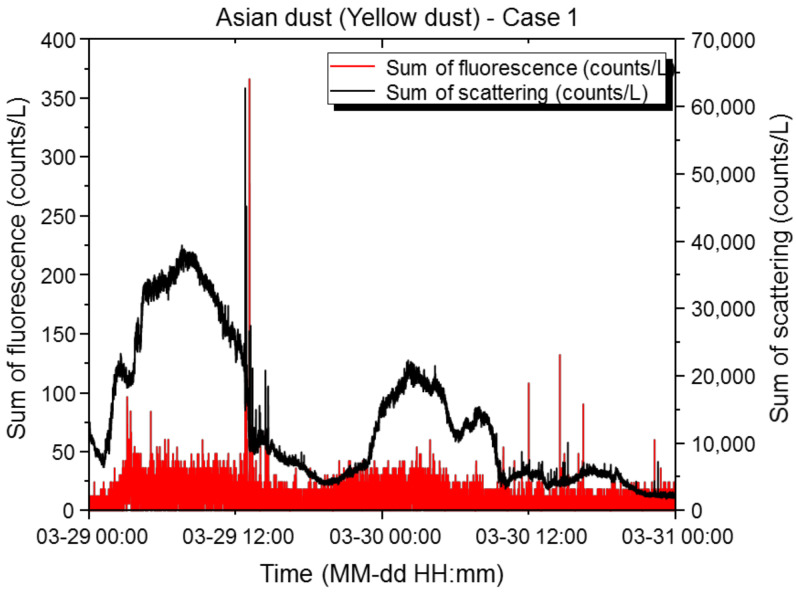
Results of light scattering and fluorescence measurements of the biological particle detector during Asian dust. Time-dependent concentrations of scattered light and fluorescent particles in Case 1. The X-axis represents the measurement period, and the Y-axis represents the measured particle concentrations. The black line represents the scattered light sum (counts/L), and the red line represents the fluorescence sum (counts/L).

An Asian dust alert is issued when an average hourly PM_10_ concentration of ≥400 µg/m^3^ lasts for ≥2 h, and an Asian dust warning is issued when an average hourly PM_10_ concentration of ≥800 µg/m^3^ lasts for ≥2 h. In addition, an average hourly PM_10_ concentration of <400 µg/m^3^ is designated as weak Asian dust, an average hourly PM_10_ concentration of 400–800 µg/m^3^ is designated as strong Asian dust, an average hourly PM_10_ concentration of ≥800 µg/m^3^ is designated as very strong Asian dust. On 29 and 30 March 2021, a warning was issued for strong Asian dust. During the Asian dust period, the maximum scattered light sum was 62,688 counts/L, the minimum was 1844 counts/L, the mean was 13,334 counts/L, and the standard deviation was 10,469 counts/L. The maximum fluorescence sum was 366 counts/L, the minimum was 0 counts/L, the mean was 5 counts/L, and the standard deviation was 10.09 counts/L. The scattering: fluorescence ratio (%) was 0.08, and the relative concentration was exceeded 216 times, with the final alarm issued 0 times. Figure 7 shows the effective number of light scattering and fluorescent particles versus time at the basic sensitivity setting.

In spring, which has a large diurnal temperature range, the air on the surface is cold and the air on the ground is warm. Asian dust in the atmosphere does not rise high but settles down; therefore, it is detected more often. The main components of Asian dust are earth dust and minerals [18]. Compared to the days without an Asian dust warning in March, the mean scattered light sum was 4-fold higher and the average fluorescence sum was 0.9-fold higher. As the concentration of the large PM_10_ particles increased due to Asian dust, the number of large light scattering particles detected by the biological particle detector also showed an increasing trend. However, the fluorescence index was very low, at approximately 0.0007, and an increase or decrease in light scattering particles was not related to changes in the fluorescent particles, due to the influence of the Asian dust components. During the Asian dust period, the number of effective biological particles did not reach the upper limit, and the reference variable for the fluorescence indexes of the scattered light sum ranges was set appropriately (based on the highest concentration range of 12,000 to 36,000 counts/L), issuing no alarms. Because the alarm was issued with the original settings, the minimum range of the scattered light weight was adjusted from 3000 counts/L to 180 counts/L and the maximum range was changed from 60,000 counts/L to 12,000 counts/L, in order to increase the weight range at high concentrations. Therefore, in a high-concentration situation (such as Asian dust), the weight for a wider range of particle concentrations was considered to be 0. As the confidence interval was widened by changing the reliability of the relative concentration from 80% to 90%, more confidence intervals were included in the population mean, which reduced the number of false alarms. During the Asian dust period, it is necessary to maintain basic or low sensitivity in order to reduce false alarms caused by the high background of light scattering particles.

#### 3.1.5. Water Waves

**Figure 8:**
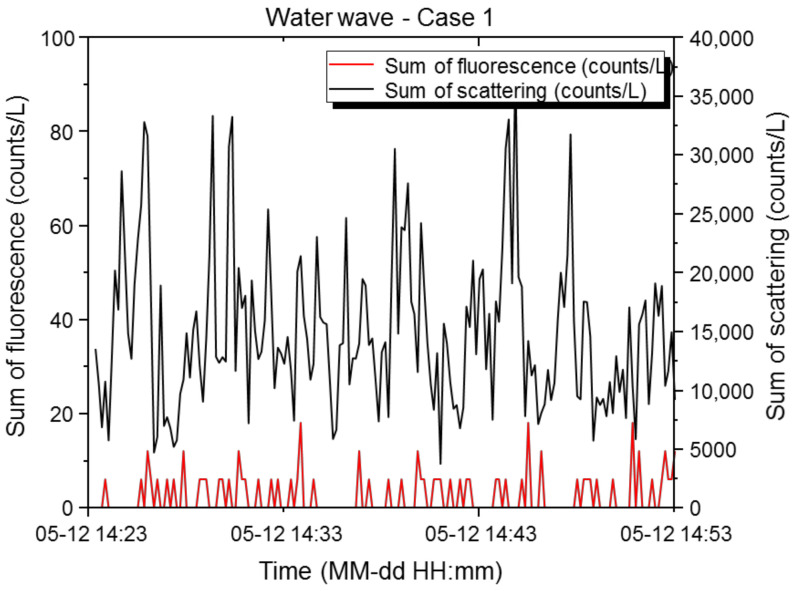
Results of scattered light and fluorescence measurements of the biological particle detector at a beach with crashing waves. Time-dependent concentrations of light scattering and fluorescent particles in Case 1. The X-axis represents the measurement period, and the Y-axis represents the measured particle concentrations. The black line represents the scattered light sum (counts/L), and the red line represents the fluorescence sum (counts/L).

**Figure 9:**
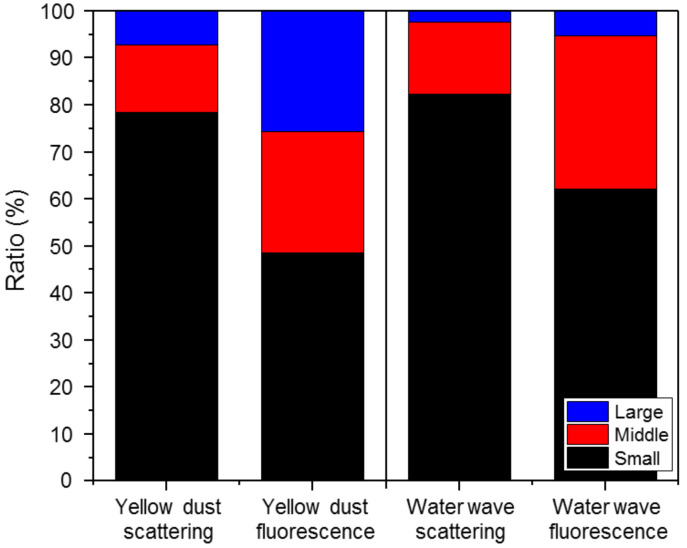
Comparison of the ratios of small, medium, and large light scattering and fluorescent particles between different environments (Yellow dust sand and a beach with crashing waves). The Y-axis represents the particle concentration, and the proportions of small, medium and large particles are indicated in black, red and blue, respectively.

With water waves, the maximum scattered light sum was 37,644 counts/L, the minimum was 3726 counts/L, the mean was 14,807 counts/L, and the standard deviation was 6677 counts/L. The maximum fluorescence sum was 18 counts/L, the minimum was 0 counts/L, the mean was 2 counts/L, and the standard deviation was 3.99 counts/L. The scattering: fluorescence ratio (%) was 0.03, and the relative concentration was exceeded 0 times, with the final alarm issued 0 times. Figure 8 shows the effective number of light scattering and fluorescent particles versus time at the basic sensitivity setting. The fine water droplets generated by waves and the scattering of sand on the beach were measured, with continuous increases and decreases in the particle numbers observed every min. This pattern coincided with the intervals of waves crashing on the beach, and the scattered light sum showed a particularly consistent trend. The scattered light sum increased rapidly a few seconds after a surge. The proportions of small, medium, and large scattered light signals during the wave periods were compared with the data obtained during the Asian dust period (Figure 9). During the Asian dust period, the proportion of scattered bands was higher than that found with water waves, as the abundance of the large PM_10_ particles increased. The proportion of small light scattering particles was higher with water waves, as they created fine water droplets as they struck the beach. Furthermore, the fluorescence characteristics followed the scattered light characteristics in both the Asian dust and water wave environments. As water waves were little influenced by other variables or emissions from vegetation, the proportion of small scattered light particles from fine water droplets was high, as was the proportion of small fluorescent particles. The biological particle detector used in this study provided accurate measurements in various environments. During the water wave period, the number of effective biological particles did not reach the upper limit of effective biological particles, and the reference variable for the fluorescence index of the scattered light sum ranges was set appropriately (based on the highest concentration range of 12,000–36,000 counts/L), issuing no alarms. Compared to the rainfall situation, fewer biological particles were emitted from the waves, with stronger re-emission from a sandy surface than from a vegetation surface, thereby issuing no alarms. Therefore, the biological particle detector is operated at the basic sensitivity setting with water waves.

### 3.2. Optimizing the Sensitivity of the Biological Particle Detector

Finally, the basic parameters of the detection algorithm were optimized for the South Korean environment by analyzing light scattering and fluorescent particles in various climates. By comparing the parameters of the detection algorithm with the set parameters established in previous studies (which did not consider the particle characteristics of each climate), improved results were obtained. A total of five variables were changed, and the developing yeast particles sequentially increased to 20 ACPLA, 38 ACPLA, 63 ACPLA, 83 ACPLA, 126 ACPLA, 167 ACPLA, and 289 ACPLA. The variables were trained using MATLAB software with the same background used in a previous study [12]. The main findings from the parameter optimization are described below:

1. The threshold value for the fluorescence index was determined according to the scattered light and fluorescence sums of the final background data. Based on the scattered light sum range, the reference variable for the fluorescence index of the lowest concentration was changed to x ≤ 1000, as follows: x ≤ 500, 500 < x ≤ 2000, 2000 < x ≤ 6000, and 6000 < x ≤ 10,000. Because the reference variable for the scattered light sum was changed to encompass a larger range, the fluorescence index in a very clean environment was judged more conservatively to reduce false alarms.
2. As the most important variable in the alarm, the fluorescence index is set to compare the sum of fluorescence (biological particles) with the scattered light sum. When changing the reference variable of the scattered light sum, the fluorescence index was also newly determined. Based on the range of each of the five levels, the fluorescence index was set at 0.016, 0.0032, 0.001, 0.0005, and 0.0002. It was set to a higher value than before to reduce false alarms, which was intended to minimize false alarms in a normal environment. The alarm rate was learned by setting different fluorescence indexes (compared to the reference variable) for the scattered light sum obtained with 6300 h of data measurements by the biological particle detector during the experiment, reflecting the characteristics of particles in various environments. The sensitivity of the biological particle detector was developed to ensure flexible adjustments through the same process for a new atmospheric environment.
3. With the algorithm, the criteria for assigning scattered light and fluorescence weights were set as follows. The weight was added to the relative concentration, initial alarm criteria, and compared with the set value for the relative concentration, initial alarm criteria. If the calculated value exceeded the set criteria, then an alarm was issued. Thereafter, the existence of biological particles was determined based on several variables, and in particular, false detections were prevented by checking whether the atmospheric particle state contained a certain level of fluorescence through the above-described fluorescence index criteria. The optimized value allowed the size that previously only received a weight of −1 to obtain a weight of −1 to −0.5. Furthermore, each reference value was set to have a higher reference value as the scattered light sum decreased. Therefore, when the scattered light range was x ≤ 30 or the fluorescence range was x ≤ 1, a weight of −1 was assigned. When the scattered light range was 0 < x < 2000 or the fluorescence range was 1 < x < 5, a weight of −0.5 was assigned. When the scattered light range was x ≥ 2000 or the fluorescence range was x ≥ 5, a weight of 0 was assigned. In the low range of the scattered light sum, an alarm could be issued even with a small number of biological particles, enabling the detector to be operated even in special situations, such as rain. Through this process, the optimized detection algorithm did not simply count biological particles, but converted the acquired signals into several variables and used them in a complex way to determine the presence or absence of biological particles.
4. As the confidence interval was widened by changing the relative concentration reliability from 80% to 90%, the wider confidence intervals included the population mean to prevent the number of effective biological particles from exceeding the upper limit, thereby reducing false alarms.

**Figure 10:**
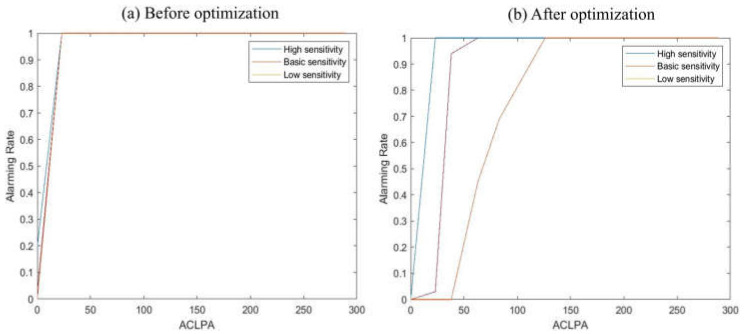
Sensitivity graph of the biological particle detector before and after applying the optimized algorithm that learned particle changes occurring in the South Korean environment.

Figure 10 shows sensitivity graphs, before and after optimization. A comparison was conducted in the general atmospheric environment, and particle concentrations ranging from a minimum of 20 ACPLA to a maximum of 290 ACPLA were considered

With the sensitivity graph before optimization, no differences were found between low, basic, and high sensitivity, and no meaningful data were obtained by adjusting the sensitivity. Even at the minimum concentration of 20 ACPLA, the alarm rate was 100% in each case, with no adaptability to various environments. However, after learning the South Korean environment and optimizing the ability to identify significant biological particles, the high sensitivity remained similar to the sensitivity before optimization while having a 100% alarm rate at a minimum concentration of 20 ACPLA, which could be used for detection and proactive actions in a clean environment. The basic sensitivity was set more conservatively than before, having a 3% alarm rate at 20 ACPLA and a 100% alarm rate at 63 ACPLA. The low sensitivity was also set more conservatively than before, having a 0% alarm rate at 20 ACPLA and a 100% alarm rate at 126 ACPLA. These optimized settings can be used to prevent the waste of military power due to indiscriminate warnings caused by biological particles that do not represent biological threats, in rainy weather or in highly polluted environments. When the concentration of biological particles was above 126 ACPLA (both before and after optimization), an alarm occurred 100% of the time, as the number of measurable fluorescent particles was sufficiently high. After optimization, the reliability of the biological particle detector when operating in special environments such as rainfall, snow, fog, mist, Asian dust, and water waves was improved, and the sensitivity could be adjusted flexibly. 

Recently, trends of local particles and their influence under various climatic conditions, such as humidity and temperature, were studied [6,16,18]. Because it is difficult to investigate fluorescent particles, there are very few papers on the subject matter. To overcome this limitation, we developed a biological particle detector and analyzed fluorescent particles for the first time in Korea. It has great significance in not only analyzing but also optimizing the developed device so that it can be operated in a Korean environment. Nevertheless, because climatic conditions are uncertain and can change at any time, it is necessary to continuously observe whether bio-particles are detected by continuously operating the monitors over long periods.

## 4. Conclusions

With the aim of optimizing the previously developed inductive fluorescence-based biological particle detector to suit the climatic conditions in South Korea, we entered simple variables generated by scattered light and fluorescence signals of particles analyzed by the biological particle detector into the algorithm and observed their complex interactions. To optimize an environmentally adaptive detection algorithm that enabled effective judgments in various situations, experiments and analyses were conducted in various climatic environments, such as rainfall, snow, Asian dust, and water wave conditions, with different locations and periods. With no prior learning about the South Korean environment, the algorithm reacted sensitively to particle distribution changes according to the climate, and over 100 false alarms were issued in 16 h. By optimizing five variables through learning, the false detection rate was decreased by 99.98% (versus no machine learning in the South Korean environment), and it was set to issue 0 alarms in situations where an alarm was unnecessary.

This study was meaningful in that it attempted to measure bioaerosols in real-time (which has become an issue recently) and that the sensitivity of the biological particle detector was flexibly adjusted to increase its reliability to suit the South Korean environment and prevent the waste of military force. The results of this study are expected to contribute to preventing various threats by finding meaningful biological weapons data with reference to the background and then producing specific weather data independently to simulate biological warfare in various environments.

## Figures and Tables

**Table 1:** Measurement period and climatic conditions in each case.

Section Title	Case Number	Measurement Period	Measurement Time (h)	Climate	Time in the Climate
3.1.1 Rainfall	Case 1	2 April 2021 16:27–6 April 2021 16:01	96	Heavy rain (maximum 5 mm)	3 April 2021 10:00–4 April 2021 09:00, Maximum rainfall at 15:00
	Case 2	12 April 2021 13:20–13 April 2021 13:52	26	Light rain (maximum 3.4 mm)	12 April 2021 13:00–13 April 2021 05:00, Maximum rainfall at 19:00
3.1.2 Snowfall	Case 1	2 January 2021 21:00–11 January 2021 12:00	112	Heavy snow (maximum 7.6 cm)	6 January 2021 22:00–11 January 2021 12:00, Maximum snowfall at 7 January 2021 04:00
	Case 2	16 February 2021 14:06–18 February 2021 13:26	71	Light snow (maximum 5.7 cm)	16 February 2021 14:00–18 February 2021 13:00, Maximum snowfall at 16 February 2021 16:00
3.1.3 Fog and mist	Case 1	5 March 2021 00:00–23:59	24	Morning fog and mist	5 March 2021 00:00–19:00
	Case 2	27 March 2021 00:00–29 March 2021 10:16	58	Afternoon fog and mist	27 March 2021 14:00–28 March 2021 09:00
3.1.4 Asian dust	Case 1	29 March 2021 00:00–30 March 2021 23:59	48	Asian dust warning (PM_10_ maintained above 800 μg/m^3^ for 2 h)	29 March 2021 00:00–30 March 2021 23:59
3.1.5 Water wave	Case 1	12 May 2021 14:23–14:53	0.5	Light wave	12 May 2021 14:23–14:53
**Abbreviation:** PM_10_: particles with diameters less than 10 µm					

## Data Availability

Not applicable.
